# Isolation and identification, genome-wide analysis and pathogenicity study of a novel PRRSV-1 in southern China

**DOI:** 10.3389/fmicb.2024.1465449

**Published:** 2024-09-11

**Authors:** Huirui Xu, Yongsheng Xie, Kehui Deng, Dongsheng He

**Affiliations:** ^1^Guangdong Provincial Key Laboratory of Zoonosis Prevention and Control, College of Veterinary Medicine, South China Agricultural University, Guangzhou, China; ^2^College of Life Science and Resources and Environment, Yichun University, Yichun, Jiangxi, China; ^3^Zhaoqing Branch Center of Guangdong Laboratory for Lingnan Modern Agricultural Science and Technology, Zhaoqing, China

**Keywords:** PRRSV-1, virus isolation, viral supernatant, genome analysis, pathogenicity

## Abstract

Porcine reproductive and respiratory syndrome virus (PRRSV) has caused severe economic losses to the global swine industry. In recent years, the incidence of PRRSV-1 has been gradually increasing in China, but there are still few studies on it. In this study, clinical samples for PRRS virus isolation were collected from a pig farm in South China in 2022. We effectively isolated a strain of PRRSV utilizing PAM cells and demonstrated its consistent transmission capability on Marc-145 cells. The isolated strain was confirmed as PRRSV-1 by RT-qPCR, IFA, electron microscopy, etiolated spot purification and whole genome sequencing, the strain was named GD2022. The length of GD2022 genome is 15058nt; Based on the genome-wide genetic evolutionary analysis of GD2022, the strain was classified as PRRSV-1. Further genetic evolutionary analysis of its ORF5 gene showed that GD2022 belonged to PRRSV-1 subtype 1 and formed an independent branch in the evolutionary tree. Compared with the sequence of the classical PRRSV-1 strain (LV strain), GD2022 has several amino acid site mutations in the antigenic region from GP3 to GP5, these mutations are different from those of other PRRSV-1 strains in China. Recombination analysis showed no recombination events with GD2022. In addition, piglets infected with GD2022 displayed clinical respiratory symptoms and typical pathological changes. In this study, a strain of the PRRSV-1 virus was isolated using both PAM cells and Marc-145 and proved to be pathogenic to piglets, providing an important reference for the identification, prevention, and control of PRRSV-1.

## 1 Introduction

Porcine Reproductive and Respiratory Syndrome (PRRS) is a highly contagious disease significantly impacting the swine industry, primarily causing reproductive disorders in pregnant sows and respiratory symptoms in pigs of all ages (An et al., 2011; VanderWaal and Deen, 2018; Yuzhakov et al., 2017). PRRSV is a single-stranded RNA virus that belongs to the *Arterivirdae* family of the order *Nidovirales* (Guo et al., 2018). The viral particles were spherical, with a diameter of approximately 60-80 nm (Rajkhowa et al., 2022). The genome, approximately 15.1-15.5 kb in length, contained 11 open reading frames (ORFs): ORF1a, ORF1b, ORF2a, ORF2b, ORF3, ORF4, ORF5, ORF5a, ORF6, ORF7, and NSP2(TF) (Conzelmann et al., 1993; Fang et al., 2012; Lunney et al., 2016; Xu et al., 2021). ORF1a and ORF1b make up 75% of the genome and encode two long polypeptides (PP) of two polyproteins, pp1a, and pp1ab (den Boon et al., 1995). Translation of ORF1a produces the pp1a polyprotein, and expression of ORF1b is shifted through the ribosomal frame resulting in the formation of the large pp1ab polyprotein (Kappes and Faaberg, 2015). The pp1a and pp1ab polyproteins are cleaved and processed by the protease encoded by ORF1a to release 13–16 nonstructural proteins (Snijder et al., 1996; Wang et al., 2013). The polyproteins are co-translationally and post-translationally processed into at least 16 distinct nonstructural proteins (nsp) via the RFSs and 4 virally encoded proteinases including papain-like cysteine proteinases 1α (PLP1α; nsp1α), PLP1β (nsp1β) and PLP2 (nsp2), and the serine proteinase (SP; nsp4) (Kappes and Faaberg, 2015; Snijder et al., 1996). PLP1α and PLPβ function to cleave the nsp1α↓nsp1β and the nsp1β↓nsp2 junction, respectively; PLP2 is responsible for the cleavage of the nsp2↓nsp3 junction and the main SP processes all remaining nsp products (nsp3–12) (Han et al., 2009; Kappes and Faaberg, 2015; Snijder et al., 1996). ORF2a, ORF2b, and ORF3-7 encode structural proteins, including six membrane-associated structural proteins, namely glycoprotein GP2a, small envelope protein E, glycoproteins GP3, GP4, GP5, membrane proteins (M proteins), and nucleocapsid proteins (N proteins) (Kappes and Faaberg, 2015; Lunney et al., 2016; Meng et al., 1995). NSP2(TF): a short transframe (TF) ORF in the nsp2 region, produces two Nsps: Nsp2N and Nsp2TF (Fang et al., 2012; Yim-Im et al., 2023).

Based on differences in genome sequence and antigenic characteristics, PRRSV is divided into two species: PRRSV-1 (European-type) and PRRSV-2 (North American-type), which share only 50–70% nucleotide sequence identity (Brinton et al., 2021). Based on the ORF5 gene sequence and analysis of the global PRRSV classification system, PRRSV-1 is commonly divided into four subtypes: subtype 1, subtype 2, subtype 3, and subtype 4 (Balka et al., 2018).

PRRSV-1 first appeared in European countries and has gradually been reported in Asia, the Americas, and elsewhere (Chen et al., 2011; Wensvoort et al., 1991). PRRSV-1 was detected in China in 1997 (Zhang et al., 2020). Since then, it has been reported in small numbers throughout the country. To date, PRRSV-1 has emerged and become widespread in China, posing a serious threat to the swine industry. PRRSV-1 has been reported in more than 20 provinces in China and is widely distributed in the central, northern, southern, eastern, northeastern and southwestern regions (Chen et al., 2011; Flay et al., 2022; Gao et al., 2017; Lin et al., 2020; Liu et al., 2017; Zhou et al., 2015). China imports breeding pigs mainly from the United States, Denmark and France, and PRRSV-1 has been detected in all of these countries (Dewey et al., 2000; Frossard et al., 2013; Kvisgaard et al., 2013, 2020; Renson et al., 2017; Ropp et al., 2004). Samples collected in Uruguay from 2014 to 2016 confirmed that PRRS had been circulating in the country as early as 2011 (Ramos et al., 2018). Testing in the U.S. Midwest detected wild-type PRRSV in 42% of pig herds (Angulo et al., 2023). In Scotland, PRRSV prevalence rates in pig farms were 45.6%, 47.8%, and 45.4% in 2006, 2013, and 2018, respectively (Correia-Gomes et al., 2022). Multiple studies have illustrated the widespread prevalence and evolution of PRRSV across different regions, highlighting the need for China to continuously monitor the virus’s spread and evolution to effectively manage potential outbreaks. In addition, recombinant strains of two vaccine strains have been reported in France, and a PRRSV-1 strain with a similar recombination pattern was subsequently reported in China (TZJ21134) (Sun et al., 2022). However, PRRSV-1 modified live vaccine (MLV) is prohibited from use in China. Thus, the recombinant vaccine strains emerging in China are likely introduced via imported breeding stock. All PRRSV-1 strains currently isolated in China belong to subtype 1. PRRSV-1 in China can be further classified into four subgroups, including BJEU06-1-like strains, Amervac-like strains, HKEU16-like strains, and NMEU09-1-like strains (Chen et al., 2017). Recent studies indicate that BJEU06-1-like PRRSV-1 strains are more widespread in China than other subgroups, suggesting they have become predominant in certain regions (Sun et al., 2023).

In this study, a novel PRRSV-1 strain was successfully isolated and shown to be pathogenic to piglets. The findings contribute to understanding the genetic evolution of PRRSV-1 strains and offer a valuable scientific basis for epidemic prevention and control.

## 2 Materials and methods

### 2.1 Collection of clinical samples and samples processing

A pig farm with 2,000 sows experienced suspected PRRSV-related reproductive issues for four months in South China in 2022. The sows exhibited reproductive disorders such as abortions, stillbirths, and mummified fetuses, with an incidence rate of about 40%. The weaned piglets showed signs of rough hair coats and respiratory symptoms, with an overall mortality and culling rate of approximately 50%. Aseptic collection of each tested sample included the serum, lung tissue, spleen tissue, and nasal swabs of the piglet. Pathology samples were homogenized and added 3ml 0.1 M phosphate-buffered saline (pH 7.4), frozen and thawed three times, then centrifuged at 12,000 × g for 10 min at 4°C, filtered for sterilization twice consecutively with 0.22 μm disposable filter membranes. The samples were then stored at −80°C for preservation.

### 2.2 Viral RNA extraction

Total RNA was extracted from the samples using the RaPure Viral RNA/DNA Kit Viral Total Nucleic Acid Rapid Extraction Kit (YuBo, Shanghai, China) according to the manufacturer’s instructions and immediately stored at −20°C until use. cDNA was synthesized using the HiScript III 1st Strand cDNA Synthesis Kit (+gDNA wiper) Reverse Transcription Kit (Vazyme, Nanjing, China) according to the manufacturer’s instructions.

### 2.3 Primer design and sample RT-qPCR detection

Based on published PRRSV sequences, primers specific to conserved regions of the genome (Supplementary Table 1) were designed for RT-qPCR detection of PRRSV using Oligo 7 software. Other primers for pathogen detection were quoted from the literature or national standards. RT-qPCR amplification was performed using Porcine Reproductive and Respiratory Syndrome Type 1 and Type 2 Multiplex Real-Time PCR Assay Kits (IDEXX, USA) according to the manufacturer’s instructions. The amplification volume was 25 μL, and the amplification conditions were as follows: 50°C for 30 min and 95°C for 1 min for the RT reaction, followed by 45 cycles of amplification at 95°C for 30 s, 60°C for 30 s, with a final cool at 35°C for 30 s.

### 2.4 Virus isolation and identification

The supernatant from the positive samples detected by RT-qPCR was diluted and inoculated with PAM cells and Marc-145 for virus isolation. The virus was blindly transmitted for 5–10 generations. The 10th-generation viral supernatant of the isolate was inoculated into MA104, 3D4/21, ST, PK15 and Vero cells to determine the adaptability of the isolate to different cells.

To detect PRRSV via IFA (indirect immunofluorescence Assay). PRRSV-infected and mock-infected cells were fixed with paraformaldehyde for 10 min and then permeabilized by 0.5% Triton X-100 in PBS for 15 minutes. After rinsing with PBS for three times, cells were blocked with 3% BSA for 1 hour. Then, cells were incubated with the PRRSV N protein (Qianxun, China) overnight at 4°C. Following 3 times washes with PBS, cells were incubated with the HyperFluor 488 Goat Anti-Mouse IgG (H+L) Antibody (APExBIO) for 1 hour. Images were captured and processed using an inverted fluorescence microscope. Electron microscopy was employed to further characterize the viral samples. We loaded 20 μL of the virus suspension onto a carbon-coated copper grid using a pipette. The sample was incubated for 5 minutes, and excess liquid was removed with filter paper. The grid was then stained with 2% phosphotungstic acid for 2 minutes. After air drying at room temperature, the grids were examined under a transmission electron microscope (TEM), and images were captured.

The virus, after 10-fold serial dilution, was inoculated onto a monolayer of Marc-145 cells. After 2 hours, the viral supernatant was removed, and 2% low-melting-point agarose was added as an overlay to restrict viral spread. Once plaques formed, they were stained with neutral red, and single plaques were selected for virus amplification. Repeating the amplification process three or more times yielded purified virus. To determine the infectious titer of PRRSV isolates, the 10th-generation viral supernatant was collected, and a TCID_50_ assay was performed. The resulting titer of the virus was calculated by the Reed-Muech method.

The 10th-generation viral supernatant successively passaged on PAMs cells and Marc-145 cells were amplified using the primers (Supplementary Table 1) and the 2 × Phanta Max Master Mix High Fidelity Enzyme Kit (Vazyme, China) according to the manufacturer’s instructions. The products were subjected to Agarose gel electrophoresis (AGE) and purified using the FastPure Gel DNA Extraction Mini Kit Gel Recovery Kit (Vazyme, China) following the manufacturer’s instructions. The purified products were sequenced (Tsingke, Ltd. Guangzhou, China). The sequencing results of ORF5 genes were compared with existing database data using the BLAST function in NCBI.

To investigate the effect of different multiplicities of infection (MOI) on the growth of PRRSV-1 in Marc-145 cells, we infected cells with MOI = 0.02 and MOI = 0.002 in triplicate. After 2 hours of incubation, the medium was discarded and replaced with 3 mL of DMEM maintenance solution. Viral supernatant was collected every 24 h and frozen at −80°C, with three samples taken at each time point. After 7 days, we determined the virus titer and drew a growth curve using GraphPad Prism 9 software. The PRRSV-1 strain isolated from Guangdong province in 2022 was designated as GD2022.

### 2.5 Complete PRRSV genome sequencing and biological analysis

Representative strains were selected from the PRRSV-1 genome sequences available in the GenBank database (Supplementary Table 2), and 10 specific primer pairs were designed using Premier 5.0 software. The designed primers (Supplementary Table 3) were applied to amplify the whole genome sequence of GD2022. The PCR product was purified and cloned into Blunt-Zero cloning vector (Vazyme, China) and then transferred into DH5α receptor cells. After amplicons were cloned into plasmids, the positive plasmid was sequenced (Tsingke, China) to achieve complete sequencing of the GD2022. Finally, the whole genome sequences of GD2022 have been deposited in the GenBank database under accession number OQ606399.

### 2.6 Sequence analysis

The sequences of ten overlapping fragments from GD2022 were assembled into full-length genome sequences using DNAstar-SeqMan software (DNASTAR, Madison, WI). The primary genome sequences of GD2022 and the full-length genome sequences of 68 reference strains (Supplementary Table 2) were analyzed for deletions and mutations in nucleotide sequences and encoded amino acid sequences using DNAstar-MegAlign7.1 (v7.1, Borland, Scotts Valley, CA). Multiple sequence alignments were performed using ClustalW in MEGA7.0 software (v7.0, Tempe, AZ). The phylogenetic evolution tree based on the whole genes and ORF5 genes was constructed in MEGA 7.0 by the neighbor-joining method with a bootstrap value of 1,000 replicates. The encoded amino acid sequences were analyzed for antigenic deterministic clusters using the NovoFocus online peptide antigen design tool. Then, the full-length genome sequence recombination analysis of GD2022 was performed using simplot3.5.1(v3.5.1, JHK University, Baltimore, MD) and RDP4.0 software.

### 2.7 Pathogenicity analysis

Ten healthy 28-day-old Yorkshire piglets (negative for PRRSV virus antigen, antibody, and other pathogens) were purchased from Guangdong Jiada Agriculture and Animal Husbandry Company. Ten piglets were randomly divided into the following groups: Group 1 (challenge group): 5 piglets were inoculated with 4 ml virus GD2022 (TCID_50_ = 10^5.5^/ml) by nasal drip + intramuscular injection; Group 2 (control group): 5 piglets were inoculated with 4 ml DMEM by nasal drip + intramuscular injection. The clinical presentations, mental status, eating, pig death, and rectal temperatures of the inoculated piglets were recorded daily. Measure the weight of piglets before and after virus inoculation and calculate the average daily gain (ADG). For detection of PRRSV antibodies, serum samples were collected on the 3rd, 6th, 9th, 12th, and 15th days post-inoculation and detected using the PRRS 2XR Virus Antibody Detection Kit (IDEXX, USA). The serum was tested for PRRSV viral content using RT-qPCR. The surviving piglets were euthanized at the end of the experiment and dissected for tissue lesions, including the heart, lungs, spleen, lymph nodes, and tonsils. At necropsy, diseased tissues were collected and fixed in 4% paraformaldehyde for histopathology analysis. Nucleic acids were extracted and reverse transcribed from the collected tissue (heart, lung, spleen, lymph nodes, tonsils), followed by quantitative PCR to detect the amount of virus in each tissue. To detect specific antigens in the infected tissues, immunohistochemistry (IHC) was employed to study a series of diseased tissues. IHC examination was carried out with the mAb-PN9cx3 antibody (GenScript Co., Ltd., Nanjing, China) to visualize the distribution of virus particles in the diseased tissues. The distribution of the virus in tissues was analyzed in conjunction with the results of quantitative fluorescent PCR and IHC.

The virus challenge experiment was approved by the Animal Ethics Committee (AEC) of the College of Life Sciences, South China Agricultural University (31 May 2023, 2023c050). All experimental procedures and animal care strictly followed the guidelines of Animal Management of South China Agricultural University.

## 3 Results

### 3.1 Results of virus isolation and identification

#### 3.1.1 RT-qPCR results of clinical samples

Lung, serum, lymph node and spleen samples were collected from 25 pigs from a suspected PRRSV-infected farm in southern China in 2022. After RT-qPCR analysis, 10 pigs were identified as PRRSV-positive, representing a positivity rate of 40% (Supplementary Figure 1A).

#### 3.1.2 Isolation and identification of viruses on cells

**FIGURE 1:**
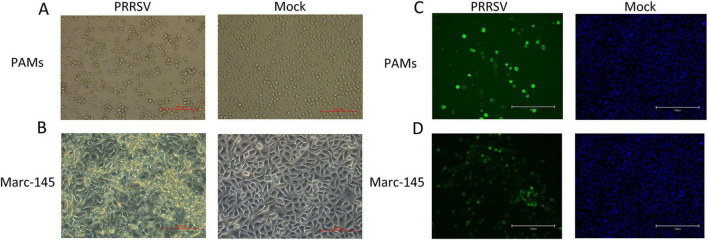
Identification of porcine reproductive and respiratory syndrome virus (PRRSV) isolates. **(A)** CPE diagram of PAMs cells infected with PRRSV isolates (left) and PAMS cells blank control (right); **(B)** CPE diagram of Marc-145 cells infected with PRRSV isolates (left) and Marc-145 blank control (right); **(C)** Result of indirect immunofluorescence assay of PAMs cells (left) and blank control (right); **(D)** Result of indirect immunofluorescence assay of Marc-145 cells (left) and blank control (right).

GD2022 strain was blindly passaged in PAMs and Marc-145 cells for ten passages, and RT-PCR was performed on the viral supernatant from the 10th passage of PAMs and Marc-145 cells (Supplementary Figure 1B). We found that the GD2022 strain could replicate in PAM and Marc-145 with CPE (Figures 1A, B) but not in the negative control group. Meanwhile, the results of cellular adaptation experiments of the virus showed that GD2022 could not replicate in MA104, 3D4/21, ST, PK15 and Vero cells. The viral supernatant of each type of cell passaged up to the 10th generation was subjected to PCR assay. The PCR results of cellular adaptation experiments of the virus showed that the viral supernatant passaged on MA104, 3D4/21, ST, PK15 and Vero cells was negative (Supplementary Figure 1C). The PCR results were consistent with the cytopathic results, indicating that the GD2022 strain could only be adapted to PAMs and Marc-145 cells.

Sequencing analysis of PCR amplification products revealed that the viral sequences of the infected PAM cells and Marc-145 cells were 100% identical, indicating that the strain could be stably transmitted on PAMs and Marc-145 cells. According to the NCBI database comparison, the identity between this strain and PRRSV-1 was 77.4∼87.0%, indicating that the isolate was a PRRSV-1 strain and was named GD2022.

**FIGURE 2:**
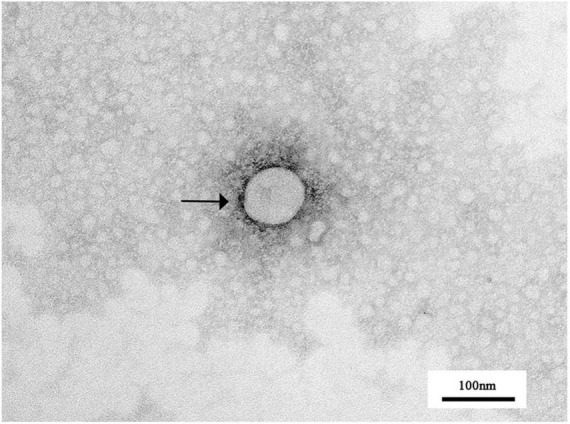
Transmission electron microscopic observation of PRRSV virions. The spherical particles marked with black arrows are PRRSV virus particles.

IFA results show that PRRSV N protein was detected in GD2022 infected PAMs (Figure 1C) and Marc-145 (Figure 1D), indicating the virus was successfully isolated from clinical samples. Under transmission electron microscopy, virus samples appeared as round viral particles with a diameter of approximately 80 nm (Figure 2).

#### 3.1.3 Virus plaque purification results and Results of TCID_50_ determination

To isolate the PRRSV strains, three viral purifications were performed. The typical PRRSV plaque pattern was observed in all Marc-145 cells infected by GD2022 strains, as confirmed by neutral red staining (Supplementary Figure 2A). Then, the 10th-generation viral supernatant was subjected to a 10-fold dilution and inoculated into a 96-well cell culture plate. Following 7-day incubation, we evaluated the number of CPE induced by GD2022 and calculated the TCID_50_ using the Reed-Muench formula. The resulting titer was determined to be 10^5.13^/mL.

#### 3.1.4 Plotting viral growth curves

To visualize the growth of the virus over time, the growth curve was plotted by measuring the TCID_50_ of the virus titer against the corresponding time points (Supplementary Figure 2B). The virus’s TCID_50_ reached its peak 72 hours after receiving the virus at MOI = 0.02, after which it began to decline. Similarly, the virus’s TCID_50_ peaked at 96 hours after receiving the virus at MOI = 0.002 and then began to decline. Under the experimental conditions, the virus proliferated most rapidly between 48 and 72 hours after virus inoculation. The virus reached the peak of TCID_50_ more quickly when the amount of virus inoculation was increased.

#### 3.1.5 Full-genome sequencing and analysis of GD2022

##### 3.1.5.1 Full-genome sequencing and structural analysis

The complete genomic sequence of GD2022 is 15058 nucleotides (nt) in length, with a 222-nt 5′UTR and a 114-nt 3′UTR, excluding the poly-A tail, which is similar in length to the published sequence of PRRSV-1. The GD2022 gene contains 8 open reading frames, including ORF1a, ORF1b, ORF2, ORF3, ORF4, ORF5, ORF6, and ORF7, with lengths of 7154bp (223 to 7377 nt), 4424bp (7359 to 11783 nt), 749bp (11,759 to 12508 nt), 794bp (12367 to 13161 nt), 548bp (12909 to 13457 nt), 605bp (13454 to 14059 nt), 521bp (14047 to 14568 nt), 386bp (14558 to 14944 nt).

##### 3.1.5.2 Homology analysis and phylogenetic analysis

**FIGURE 3:**
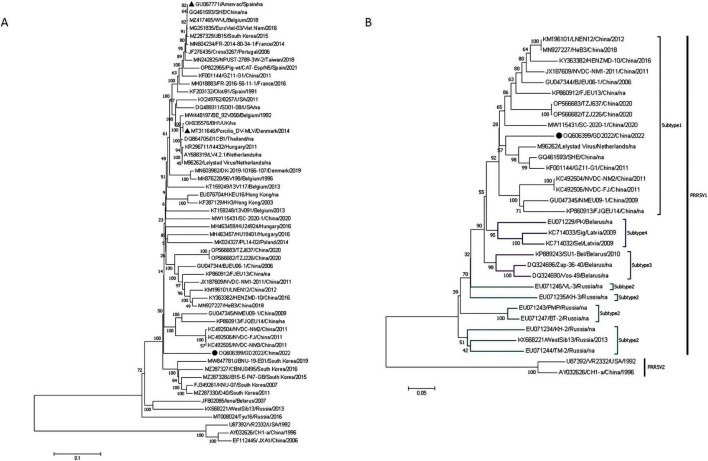
Whole genome analysis of GB2022. **(A)** GB2022 phylogenetic tree based on the whole genome sequence. **(B)** GB2022 phylogenetic tree based on PRRSV ORF5 gene. “
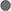
” is GB2022. “
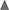
” is PRRSV-1 vaccine strain.

Homology analysis revealed that GD2022 shares the highest identity with PRRSV-1 strains ranging from 77.4% to 87.0%, with 87.0% identity to the PRRSV-1 representative strain (LV strain) and only 59.7% identity to the PRRSV-2 representative strain (VR2332 strain). The results of the full-genome phylogenetic tree showed that GD2022 was in a small branch of the PRRSV-1 and was genetically distant from the PRRSV-1 vaccine virus (Figure 3A). Genetic evolutionary analysis based on the ORF5 genes showed that GD2023 belongs to PRRSV-1 subtype 1. The GD2022 was on a separate branch of the genetic phylogenetic tree (Figure 3B).

##### 3.1.5.3 Deletion and mutation analysis and Recombinant analysis of the sequence

The amino acid sequences of the proteins encoded by GP3, GP4, and GP5 of GD2022 were analyzed in comparison with those of the domestic strain and the classical strain (LV strain) (Supplementary Figure 3), and it was found that there were three deletions in the overlapping region of the proteins encoded by GP3 and GP4 of this strain, GP3 and GP4 proteins are each missing an amino acid at positions 245aa and 65aa, respectively. One mutation occurred in the neutralizing epitope of the GP5 protein at position 33aa (D→A). Two mutations occurred in the highly variable region at the positions 56aa (D→A) and 63aa (G→D). This deletion and mutation are different from other PRRSV-1 strains in China.

We assessed the recombination using RDP4. 0 (RDP, GENCONV, MaxChi, Chimaera, BootScan, 3Seq, SiScan). No recombination breakpoints were identified from the whole genomic sequence alignment of GD2022. The above results showed that GD2022 was a novel PRRSV-1 strain.

### 3.2 Pathogenicity analysis of GD2022

#### 3.2.1 Clinical signs of piglets

**FIGURE 4:**
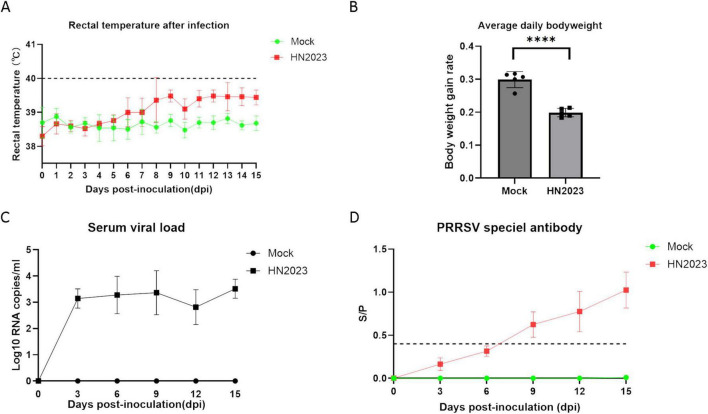
Changes in body temperature **(A)** and body weight **(B)** of piglets after challenge. Changes in virus titers **(C)** and antibody levels in the serum **(D)** of piglets after challenge.

After the challenge, the body temperature of the challenge piglets started to rise on the 6th day, and one pig was above 40°C on the 8th day. From the 6th day, the temperature of the challenge group was 1°C higher than the control group (Figure 4A). The control pigs remained normal throughout the experiment. On the third day, the challenged pigs began to exhibit anorexia. From the seventh day, the clinical signs worsened, and the challenge piglets began to exhibit respiratory symptoms such as sneezing and wheezing, along with respiratory distress. In contrast, the control group show no abnormal clinical signs. Slow weight gain of piglets in challenged group and the body weight of the challenge group was significantly lower than that of the control group (P < 0.001) (Figure 4B).

#### 3.2.2 Viremia and PRRSV antibodies

Viremia of piglets in the GD2022-infected groups was detected within 3–15 dpi. The serum virus RNA copy numbers in the GD2022-infected piglets exceeded 10^3^ copies/ml during 3-15 dpi (Figure 4C). On day 15 after the challenge, the amount of virus in blood reached the peak. No viremia was detected in the serum samples from the control group throughout the entire experimental period. The serum level of PRRSV antibodies was evaluated by ELISA. The result revealed that the antibody level showed an upward trend after challenge (Figure 4D). The antibody levels were higher than the positive threshold set by the kit from the 9th dpi and maintained a sustained upward trend. In the control group, the result was negative.

#### 3.2.3 Pathological observation after dissection

**FIGURE 5:**
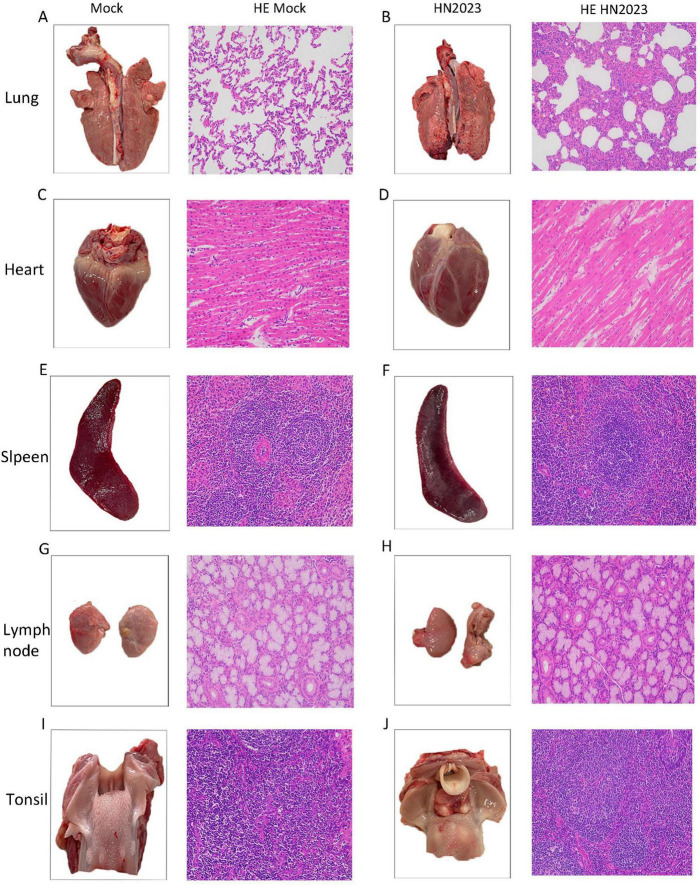
Results of pathological changes in various tissues and organs of piglets after challenge. **(A)** Lung and histopathological sections from the control group; **(B)** Lung and histopathological sections from the challenged group; **(C)** Heart and histopathological sections from the control group; **(D)** Heart and histopathological sections from the challenged group; **(E)** Spleen and histopathological sections from the control group; **(F)** Spleen and histopathological sections from the challenged group; **(G)** Submandibular lymph node and histopathological sections from the control group; **(H)** Submandibular lymph node and histopathological sections from the challenged group; **(I)** Tonsil and histopathological sections from the control group; **(J)** Tonsil and histopathological sections from the challenged group.

Piglets in the challenge group showed obvious pneumonia, with tissue consolidation and bleeding points on the lung surface (Figure 5B). The heart was flaccid and exhibited bleeding points in the myocardium (Figure 5D). The spleen exhibited jagged edges with obvious bleeding points (Figure 5F). There were no discernible lesions on the ocular view of the tonsils and lymph nodes. The control group showed normal tissue structure in the organ.

The results of pathological sections are shown in Figure 5. In the challenge group, the structure of the lung tissue of piglets was abnormal, with thickening of the lung interstitium, a significant reduction in the alveolar lumen, increased exudate and a decrease in cell number (Figure 5B). The cardiac fibers were disorganized (Figure 5D). There were no obvious abnormalities of the spleen, submandibular lymph nodes and tonsils.

#### 3.2.4 Assessment of viral loads in organs after dissection

**FIGURE 6:**
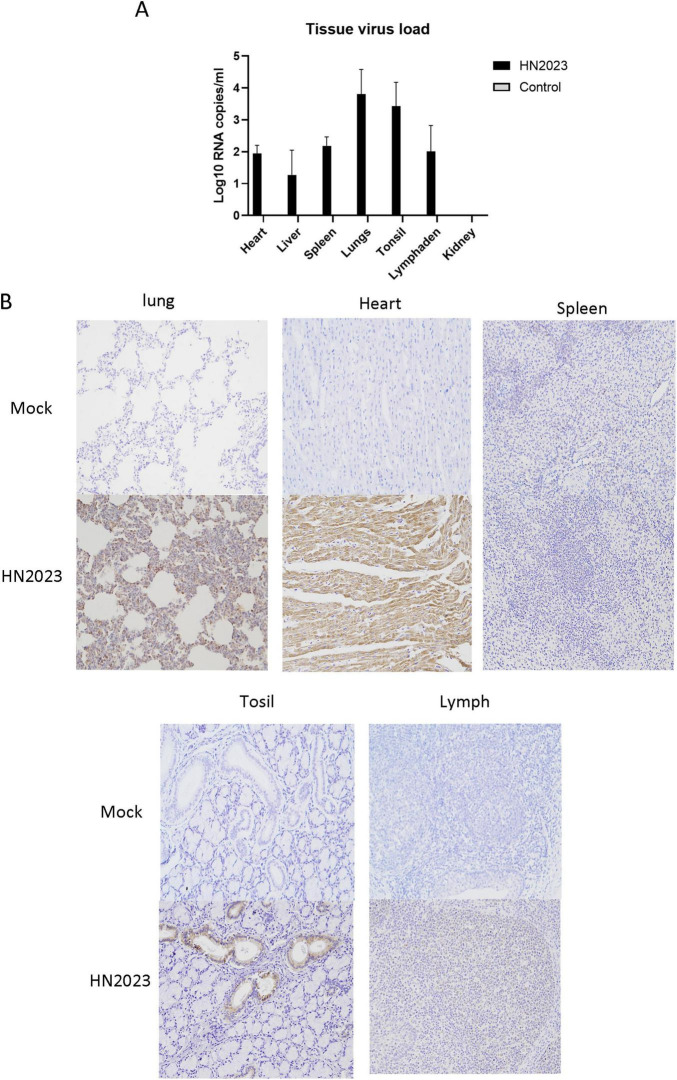
**(A)** Viral load in tissues of various organs. **(B)** Results of immunocytochemistry of histopathological sections of various organs.

Viral load in the heart, liver, spleen, lungs, kidneys, tonsils, and lymph nodes was quantified using RT-qPCR. The highest viral load was observed in the lungs and tonsils, with the kidney registering the least viral presence (Figure 6A). The results of IHC staining showed that the lungs and submandibular lymph nodes had clear positive signals (Figure 6B). In the control group, the result of RT-qPCR was negative and IHC staining showed no positive signals. In the analysis of the above results, GD2022 mainly infected lung tissue.

## 4 Discussion

Porcine reproductive and respiratory syndrome (PRRS) first emerged in China in 1996, rapidly spreading throughout the country, and causing major economic losses to the pig industry (An et al., 2007; Sui et al., 2018). In recent years, PRRSV has been reported in the United States (PRRSV-1) (Wang et al., 2019), North America (Yu et al., 2020), and Brazil (Gava et al., 2022). In addition, a study using a dataset of 82,237 PRRSV-2 ORF5 sequences from several countries found that PRRSVs with different spectrums have been reported in China, the United States, Canada, Denmark, Germany, Spain, and Mexico (Yim-Im et al., 2023). These studies indicate that PRRSV is widespread abroad, providing new insights into virus transmission and highlighting the importance of virus surveillance. The main strategy for preventing and controlling PRRS outbreaks is vaccination. However, some studies have shown PRRSV vaccines to be inefficient and ineffective (Guo et al., 2018). Nonetheless, vaccination remains the primary method of PRRSV prevention. The major commercialized vaccines currently available for the prevention and control of PRRSV include inactivated vaccines, MLVs, subunit vaccines, DNA vaccines, and virus vector vaccines (Nan et al., 2017). From the 1990s, PRRSV-1 MLV was used to prevent PRRSV-1 infection in Europe (Chae, 2021). Common PRRSV-1 MLVs used worldwide include Porcilis PRRS (MSD Animal Health), Amervac PRRS (Hipra), ReproCyc PRRS EU (Boehringer Ingelheim), Pyrsvac-183 (SYVA Laboratories), Ingelvac PRRSFLEX^®^ EU (Boehringer Ingelheim), Suvaxyn^®^ PRRS MLV (Zoetis), and Unistrain^®^ PRRS (Hipra) (Chae, 2021; Zhou et al., 2021). However, PRRSV-MLVs only provide partial protection (Charerntantanakul, 2012; Roca et al., 2012). Some studies suggest that PRRSV-MLVs have the potential to revert to virulence (Yu et al., 2021). In addition, recombination between MLVs and wild-type strains has exacerbated PRRS outbreaks (Kikuti et al., 2021; Kimman et al., 2009; Read et al., 2015). The safety issues associated with vaccines have exacerbated the challenges of controlling PRRSV-1 in China. Commercialized vaccines currently available in China to prevent PRRSV include VR-2332 (Boehringer-Ingelheim, Mannheim, Germany), CH-1R (DaHuaNong Company, Guangdong, China), JXA1-R (Guangdong Yongshun Biological Pharmaceutical Co. Ltd. Guangdong, China), TJM-F92 (Qingdao Yibang Biological Engineering Co. Ltd) and so on. In China, PRRS vaccines have mainly been developed for PRRSV-2 (Zhang et al., 2023). At present, there are problems such as low attention to PRRSV-1, lack of effective detection methods in clinical practice and insufficient stockpile of PRRSV-1 vaccine, so the prevention and control of PRRSV-1 infections are mainly based on biosecurity management measures (Sun et al., 2023).

Given that these issues persist, with increasing detection data, several studies have reported recombination and mutation in PRRSV-1 in recent years. Studies have shown that recombinant strains tend to be more virulent than their parental strains (Yu et al., 2021). Yu et al. showed that high-frequency sites of recombination were mainly concentrated in Nsp2 and GP2-GP4 (Yu et al., 2022). Other scholars used NMEU09-1, BJEU06-1, Amervac and HKEU16 to analyze whole genes of PRRSV-1 strains. They found the recombination sites were in Nsp2, Nsp3, Nsp9, Nsp10 and GP3-N (Sun et al., 2023). In our study, we isolated a PRRSV strain from a clinical sample collected in 2022 and this strain was named GD2022. Full genome sequencing showed that GD2022 is 15,058bp in length, with 87.0% identical to the PRRSV-1 representative strain (LV strain). A phylogenetic tree was constructed based on the ORF5 gene sequences, the PRRSV-1 strain could be divided into four subtypes, and the GD2022 was classified within subtype 1. Then, we used Simplot to analyze the recombination of GD2022. The results showed that no recombination occurred in GD2022. Although the strain we isolated did not undergo recombination, subsequent pathogenicity studies in piglets still showed that the strain caused mild clinical symptoms, including slow weight gain, sneezing and fever. Slow weight gain can prolong the breeding cycle, increase feed costs, reduce economic efficiency, and lead to decreased productivity. Therefore, we should not only strengthen the epidemiological monitoring of recombinant strains but also pay attention to the potential harm caused by non-recombinant strains.

In addition to recombination, mutation is another important mechanism of PRRSV evolution (Guo et al., 2018). Recent studies indicate that gene fragment mutations contribute to the development of new biological properties. These mutations can alter antigenicity and enhance pathogenicity, enabling the virus to evade the host immune response and cause more severe disease in infected pigs (Bian et al., 2017; Sun et al., 2016). Particularly, these changes include amino acid mutations in key viral proteins such as Nsp2, ORF3, and ORF5. For instance, in 2011, Chen et al. (2011) first isolated PRRSV-1 wild-type strains BJEU06-1 and NMEU09-1, which exhibited amino acid mutations in the Nsp2, ORF3, and ORF5 (Chen et al., 2011). In the same year, Wang X. et al. isolated GZ11-G1, which showed extensive amino acid mutations in proteins such as Nsp2 (Wang et al., 2016). In 2016, Zhai et al. collected multiple diseased materials from five farms in Guangdong Province, the ORF5 nucleotide sequences of several isolates showed varying degrees of mutation (Zhai et al., 2018). Compared to the classical strain of PRRSV-1 (LV strain), GD2022 in this study had three deletions in the overlapping region of the GP3 and GP4, which were each missing an amino acid at positions 245aa and 65aa respectively. One mutation was found in the GP5 protein at position 33aa (D→A). Two mutations were found in the highly variable region of the GP5 protein at position 56aa (D→A) and 63aa (G→D). Mutations in viral strains can result from adaptive variation of the virus as it spreads in different ecological environments under the influence of factors such as temperature, humidity, and host density; they can also result from host immune pressure that leads to mutations at specific amino acid sites to evade immune recognition and maintain transmission. In addition, viruses often make replication errors during replication, leading to further amino acid mutations. The mutation of GD2022 in the highly variable region ORF5 distinguishes it from other PRRSV-1 strains in China. This finding maybe a biomarker for novel PRRSV-1 isolates in China.

As national quarantine policies have tightened, the detection rate of PRRSV has annually increased, with a notable rise in PRRSV-1 incidence in South China (Zhai et al., 2018). The increase in detection can be attributed to enhanced surveillance and stricter quarantine regulations, leading to more accurate diagnosis and understanding of PRRSV-1 cases in the region. As a result, our team isolated a novel PRRSV-1 strain, designated GD2022. We studied the genetic evolution of the strain, conducting detailed recombinant and mutation analyses, and assessing its pathogenicity. Our study has certain limitations. In piglet pathogenicity experiments, the mental status and health of the piglets should be assessed before the challenge, and during the experiment it should be ensured that all piglets are kept under the same conditions of temperature to ensure the validity of the results. Besides that, piglet weights should be measured daily at the same time to more accurately observe the difference in daily weight gain between piglets in the attack group and the control group. In recent years, PRRSV-1 has been isolated mainly from PAM cells, and there are fewer reports of successful isolation using cell lines. In this study, a PRRSV-1 strain was successfully isolated, propagated, and purified using the Marc-145 cell line. Since PAM cells are expensive and difficult to obtain, the successful isolation of the virus in cell lines not only simplified the research process but also provided valuable raw material for subsequent vaccine studies. Our findings establish a significant theoretical foundation for future PRRSV research, with implications for the development of PRRSV-1 vaccines. Despite the lower pathogenicity of PRRSV-1, our results indicate that clinical symptoms and transmissibility occur post-infection. The current underestimation of PRRSV-1 in China may facilitate its spread, posing economic risks to the swine industry. Thus, our study provides critical insights for the control and prevention of PRRSV infections.

## Data Availability

The datasets presented in this study can be found in online repositories. The names of the repository/repositories and accession number(s) can be found in this article/Supplementary material.
