# Supplementary material for: Isolation and identification, genome-wide analysis and pathogenicity study of a novel PRRSV-1 in southern China
Source: Front Microbiol. 2024 Sep 11;15:1465449. doi: 10.3389/fmicb.2024.1465449 (PMC11422217; doi:10.3389/fmicb.2024.1465449)
Supplement: Supplementary file 1 [file Data_Sheet_1.pdf]

Supplementary Material

Isolation and identification, genome-wide analysis and pathogenicity study of a novel PRRSV-1 in southern China

Huirui Xu[1,3*], Yongsheng Xie[2*], Kehui Deng[1,3], Dongsheng He[1,3**]

[1] *Guangdong Provincial Key Laboratory of Zoonosis Prevention and Control, College of Veterinary Medicine, South China Agricultural University, Guangzhou, 510642, China.*

[2] *College of Life Science and Resources and Environment, Yichun University, Yichun, 336000, Jiangxi, China.*

[3] *Zhaoqing Branch Center of Guangdong Laboratory for Lingnan Modern Agricultural Science and Technology, Zhaoqing, 526238, China.*

*These authors contributed equally

**Corresponding author:

Prof. Dongsheng He.

Mailing address: College of Veterinary Medicine, South China Agricultural University, Guangzhou, 510642, China

Email: dhe@scau.edu.cn

1 Supplementary Figures and Tables

1.1 Supplementary Figures

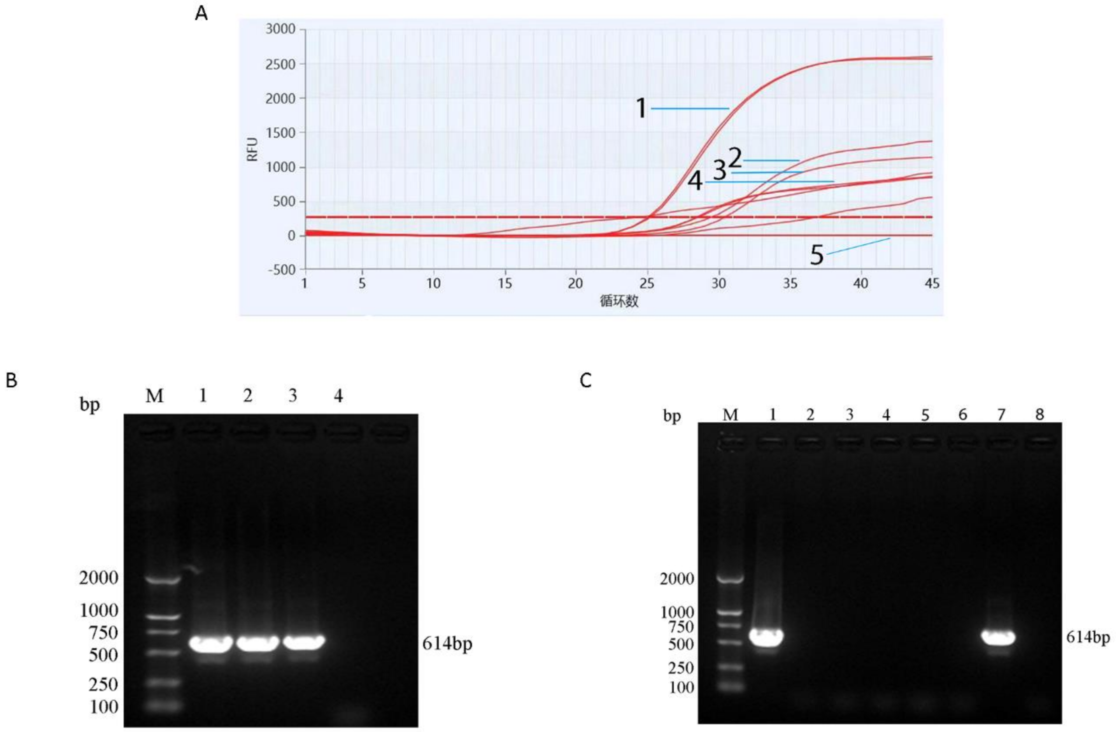

Supplement Figure 1. (A) RT-qPCR results of collected disease material (partial). 1 is a positive control (two replicates), 2-4 are clinical samples (Selected positive samples were subjected to qPCR for viral load detection); 5 is a negative control (two replicates). (B) PCR results of isolated strains**.** M is DL2000 DNA marker; 1 is 10th generation virus fluid on PAM cells; 2 is 10th generation virus fluid on Marc-145 cells; 3 is positive control; 4 is negative control. (C) Adaptation of isolated strains to PCR assays in different cells. M, DL2000 DNA marker; 1, 10th generation virus on Marc-145 cells; 2, 10th generation virus on MA104 cells; 3, 10th generation virus on 3D4/21 cells; 4, 10th generation virus on ST cells; 5, 10th generation virus on PK15 cells; 6, 10th generation virus on Vero cells; 7, positive control; 8, negative control.

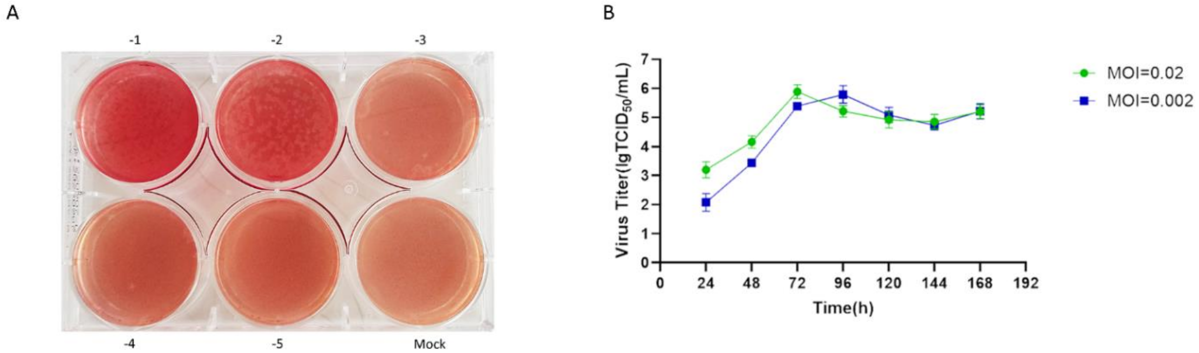

Supplementary Figure 2. (A) Isolated strain etiolated plaque purification experiments. (B) Growth curve of PRRSV-1 on Marc-145 cells

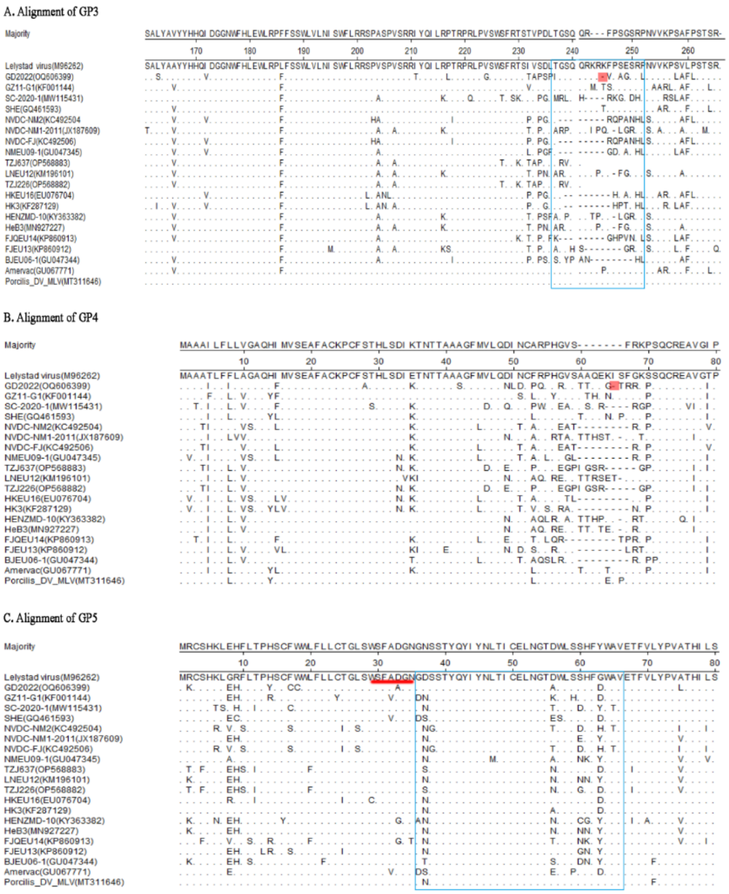

Supplementary Figure 3. Amino acid sequence comparison of GP3, GP4 and GP5 structural proteins. (A) Amino acid sequence comparison of GP3 structural proteins; (B) Amino acid sequence comparison of GP4 structural proteins; (C) Amino acid sequence comparison of GP5 structural proteins; Supplementary Tables. The deletion regions of GP3 and GP4 are marked with red boxes. The blue box regions of GP3 and GP5 amino acid sequences were hypervariable regions. The five amino acids underlined in red are neutralizing epitopes

Supplementary Table 1. The primer sequences serve to identify virulent strains and facilitate the detection of other pathogens

Primer Name	Sequence (5'-3')	Length of products(bp)	Origin
PRRSV1-JD	F: CTGCAATGAGGTGGGCTACAAC	614	Design
	R: TAACCCCTTCGAGGACGACAT		
PRRSV2-JD	F: AGGTGGGCAACTGTTTTAGC	697	Design
	R: TTTGTGGAGCCGTGCTATCA		
PEDV-JD	F: TTC GGT TCT ATTCCCGTTGATG	663	GB/T 36871-2018
	R: CCCATGAAGCACTTTCTCACTATC		
TGEV-JD	F: TTACAAACTCGCTATCGCATGG	528	GB/T 36871-2018
	R: TCTTGTCACATCACCTTTACCTGC		
PoRV-JD	F: GAAACGGAATAGCTCCACAAT	271	GB/T34756-2017
	R: GAATAATCAAATCCAGCCACC		
PRV-JD	F: CAGGAGGACGAGCTGGGGCT	217	GB/T 18641-2018
	R: GTCCACGCCCCGCTTGAAGCT		
CSFV-JD	F1: AGRCCAGACTGGTGGCCNTAYGA	671	GB/T 36875-2018
	R1: TTYACCACTTCTGTTCTCA		
	F2: TCR WCAACCAAYGAGATAGGG	272	
	R2: CACAGYCCRAAYCCRAAGTCATC		
PCV2-JD	F: CCGCGGGCTGGCTGAACTT	1154	GB/T 21674-2008
	R: ACCCCCGCCACCGCTACC		

Supplement Table 2. Information on strains referenced for primer design

Strain Name	Country	Login Number	Strain Name	Country	Login Number
PK	Byelorussia	EU071229	FJEU13	China	KP860912
Lena	Byelorussia	JF802085	PL14-02	Poland	MK024327
SC-2020-1	China	MW115431	NVDC-NM1-2011	China	JX187609
WVL	Belgium	MZ417465	LNEU12	China	KM196101
13V091	Belgium	KT159248	Sig	Latvia	KC714033
FJQEU14	China	KP860913	Sel	Spanish	KC714032
NMEU09-1	China	GU047345	VL-3	Russia	EU071246
NVDC-FJ	China	KC492506	8257	USA	KX249762
NVDC-NM2	China	KC492504	KH-3	Russia	EU071235
NVDC-NM3	China	KC492505	DK2019-10166-107	Denmark	MN603982
WestSib13	Russia	KX668221	Lelystad virus	Netherlands	M96262
HK3	Hong Kong	KF287129	Amervac PRRS	Spanish	GU067771
Tyu16	Russia	MT008024	SHE	China	GQ461593
HKEU16	Hong Kong	EU076704	14432	Netherlands	AY588319
JBNU-19-E01	South Korea	MW847781	HeB3	China	MN927227
D40	South Korea	MZ287330	PMP	Russia	EU071243
BJEU06-1	China	GU047344	CH-1a	China	AY032626
15HEN1 EU	China	KX967492	VR-2332	USA	U87392
GZ11-G1	China	KF001144	JXA1	China	EF112445
Euro Viet-03	Viet Nam	MG251835	JB15	South Korea	MZ287329
FR-2014-80-34-1	France	MN604234	CBNU0495	South Korea	MZ287237
FR-2016-56-11-1	France	MH018883	JB15-E-P47-GB	South Korea	MZ287238
Cresa3267	Portugal	JF276435	KUN-07	South Korea	FJ349261
NPUST-2789-3W-2	Taiwan	MN242825	SD01-08	USA	DQ489311
BE92V058	Belgium	MW448197	BH1	UK	OK635576
96V198	Belgium	MK876228	Porcilis DV-MLV	Denmark	MT311646
13V117	Belgium	KT159249	01CB1	Thailand	DQ864705
14432	Hungary	KR296711	TZJ637	China	OP566683
HU24924	Hungary	MH463459	TZJ226	China	OP566682
HU19401	Hungary	MH463457	HENZMD-10	China	KY363382
SU1-Bel	Byelorussia	KP889243	Vos-49	Byelorussia	DQ324690
Zap-36-40	Byelorussia	DQ324696	BT-2	Russia	EU071247

Strain Name	Country	Login Number	Strain Name	Country	Login Number
KH-2	Russia	EU071234	TM-2	Russia	EU071244
WestSib13	Russia	KX668221	Olot/91	Spain	KF203132

Supplementary Table 3. Genome-wide primer sequences of PRRSV-1 strain GB2022

Primer name	Sequence (5’-3’)	Length of products(bp)
1	F: ATGATGTGTAGGGTATTCCCCCT	2123
	R: ACTTTGATTTTTGCCAGAAGGGC	
2	F: ACGTGCCCTTGAGGCCTTG	2022
	R: GCATGACACTCCGGCGAATC	
3	F: CTCTATGGCTCCAGGTGATTGG	2197
	R: CATCAGGGATGATGGCCGG	
4	F: TCCTACAACCACATGCACAC	1961
	R: GCGGCTAGCAGTTTAAAGCCT	
5	F: GAGCAAGCCCTTGCTGGGATG	2110
	R: CACAAATCAAGTCCACAGGCG	
6	F: ATGTCCATGTGGGAGAAACTGAA	2015
	R: GTGGCTCCTTTCCAGACCATCA	
7	F: GTGTGCACCCTCACCGATG	1845
	R: AACAGGGCTTACAGGCGAAC	
8	F: GGCAACACATCATTCGAGCT	2022
	R: GAGGCTCCGAAGTCCTGGTAC	
9	F: CTTCCAGATGCAGATTGTGTTGC	689
	R: CCATGTGATCGCCCTAATTGAATAG	
10	F: GGCGCAGGAACTGCGTCGCTTTCATCCA	499
	R: TTTTTTTTTTTTTTTTTTTTTTTTTTTTT	
